# Keywords to Recruit Spanish- and English-Speaking Participants: Evidence From an Online Postpartum Depression Randomized Controlled Trial

**DOI:** 10.2196/jmir.2999

**Published:** 2014-01-09

**Authors:** Alinne Z Barrera, Alex R Kelman, Ricardo F Muñoz

**Affiliations:** ^1^Palo Alto UniversityPalo Alto, CAUnited States

**Keywords:** Internet intervention, prevention, depression, postpartum, research subject recruitment, women, Spanish speaking

## Abstract

**Background:**

One of the advantages of Internet-based research is the ability to efficiently recruit large, diverse samples of international participants. Currently, there is a dearth of information on the behind-the-scenes process to setting up successful online recruitment tools.

**Objective:**

The objective of the study was to examine the comparative impact of Spanish- and English-language keywords for a Google AdWords campaign to recruit pregnant women to an Internet intervention and to describe the characteristics of those who enrolled in the trial.

**Methods:**

Spanish- and English-language Google AdWords campaigns were created to advertise and recruit pregnant women to a Web-based randomized controlled trial for the prevention of postpartum depression, the Mothers and Babies/*Mamás y Bebés* Internet Project. Search engine users who clicked on the ads in response to keyword queries (eg, pregnancy, depression and pregnancy) were directed to the fully automated study website. Data on the performance of keywords associated with each Google ad reflect Web user queries from February 2009 to June 2012. Demographic information, self-reported depression symptom scores, major depressive episode status, and Internet use data were collected from enrolled participants before randomization in the intervention study.

**Results:**

The Google ads received high exposure (12,983,196 impressions) and interest (176,295 clicks) from a global sample of Web users; 6745 pregnant women consented to participate and 2575 completed enrollment in the intervention study. Keywords that were descriptive of pregnancy and distress or pregnancy and health resulted in higher consent and enrollment rates (ie, high-performing ads). In both languages, broad keywords (eg, pregnancy) had the highest exposure, more consented participants, and greatest cost per consent (up to US $25.77 per consent). The online ads recruited a predominantly Spanish-speaking sample from Latin America of *Mestizo* racial identity. The English-speaking sample was also diverse with most participants residing in regions of Asia and Africa. Spanish-speaking participants were significantly more likely to be of Latino ethnic background, not married, completed fewer years of formal education, and were more likely to have accessed the Internet for depression information (*P*<.001).

**Conclusions:**

The Internet is an effective method for reaching an international sample of pregnant women interested in online interventions to manage changes in their mood during the perinatal period. To increase efficiency, Internet advertisements need to be monitored and tailored to reflect the target population’s conceptualization of health issues being studied.

**Trial Registration:**

ClinicalTrials.gov NCT00816725; http://clinicaltrials.gov/show/NCT00816725 (Archived by WebCite at http://www.webcitation.org/6LumonjZP).

## Introduction

The amount of Internet-based research in recent years has increased substantially [1-3] with Internet interventions showing significant promise in both alleviating symptoms and changing behavior [2]. Internet interventions are accessible at any time from any location, can be used anonymously, and may provide a much-needed service to users who feel marginalized or stigmatized [1,2,4]. For researchers, the Internet is an effective method of data collection [5] given that it is cost-effective, efficient at recruiting large sample sizes, and provides an opportunity for sensitive topics to be examined among hard-to-reach populations [6-9].

Most Internet intervention studies have focused on depression and anxiety, but an emphasis on other health issues is gaining interest as further evidence of their effectiveness is demonstrated [2,10]. The characteristics of those who participate in Internet interventions are dependent on the target problems being addressed; however, online study samples repeatedly include a greater proportion of women than men. This trend is not surprising given that the percentage of women using the Internet has steadily increased in recent years [11], and women are reported to be the largest group of Web users searching for health information related to mental illness [12]. The higher incidence of depression among women relative to men is likely a contributing factor to the greater focus on mental health topics, such as depression, among female Internet users.

Women and women-specific health issues are well suited for technology-based interventions with perinatal women garnering much of the attention by researchers (eg, [13-17]). Currently, there are countless websites that provide information on pregnancy and postpartum health. A recent review, however, revealed that the quality of the content exhibited on websites that focused on maternal mental health was partially correct or incomplete at best, and provided few self-help tools and resources to users [18]. Thus, there is a need to develop and empirically test Internet interventions specifically designed for women that address issues that uniquely affect women, such as postpartum depression (PPD). Computer- and Internet-based methods have been used successfully to increase awareness of PPD among adolescent parents [19], to recruit and screen women at risk for PPD [20-22], or to disseminate PPD psychoeducational materials to postpartum women and clinicians, all of whom have responded positively to this platform [22,23]. Recent studies have demonstrated support for behavioral activation [24,25] and cognitive behavioral [26] Internet interventions that aim to reduce depressive symptoms among depressed postpartum women. Two recent reports describe the design, feasibility, and acceptability of Internet interventions to prevent PPD [27,28]. The study presented in this report is based on data collected as part of the Mothers and Babies/*Mamás y Bebés* Internet Project, an Internet-based, 2-condition, pilot randomized controlled trial (RCT) designed to examine the efficacy of a Web-adapted mood management prevention intervention (Mothers and Babies Course/*Curso de Mamás y Bebés*) [29]. Participants were recruited, screened, and randomized to either the mood management Internet intervention or to an information brochure [23]. Preliminary analyses from the prevention trial are currently underway.

Although the Internet offers added benefits and opportunities, there are significant concerns and barriers with recruitment to Internet-based research investigations [30]. Many of these investigations rely on traditional forms of recruitment such as face-to-face recruitment, flyers, community messages, and mass media announcements [31-33]. Earlier studies that used online recruitment strategies struggled with identifying and reaching the target population (eg, [6,34]), low rates of response [35], and possible ethical concerns with recruiting over the Internet [3]. The more common methods of online recruitment are search engine advertisements, social media sites, online forums, Web links, banners, and email lists. The chosen method of recruitment depends on the population being targeted and where their online presence is greatest. However, recent reports suggest that targeted search engine advertisements are the most successful at reaching a wide range of potential participants for Internet research and are cost-effective methods of online recruitment [6,8].

What remains unclear in the literature is how researchers should frame their Internet advertisements so that they are time-efficient and cost-effective (ie, targeted to a specific population who are most likely to enroll in the study). Graham and colleagues [36] provide a good example of delineating the process of tailoring online advertisements to recruit Latino smokers to an online smoking cessation website. As part of their process, the researchers convened a multicultural panel of experts who informed the content of banner ads that were then empirically tested among Latino smokers. The researchers found that tailoring banner ads to portray the cultural value of *familismo* (emphasis on the importance of family influences) was preferable among Spanish-speaking Latino smokers who indicated that the issue of smoking was not just about them, but also about the impact on the relationships they valued [36]. Perhaps more importantly, this study demonstrated that a behind-the-scenes exploration of key factors that influence the success of an online recruitment method is valuable and needs to be empirically tested to maximize the strength of online recruitment. Furthermore, it is recommended that researchers continually monitor the behaviors of users once they are actively engaged in the study website [37].

The current study adds to the growing body of literature describing online recruitment methods for Internet intervention studies. The primary goal was to examine the impact of Google AdWords campaigns to recruit Spanish- and English-speaking pregnant women to an Internet RCT to prevent PPD. Both the Spanish and English ad campaigns used identical keywords and were minimally modified throughout the recruitment period. Given that few Internet intervention studies have focused exclusively on pregnant women, we describe the demographic characteristics of participants who enrolled in the trial.

## Methods

### Internet Search Ads

**Figure 1:**
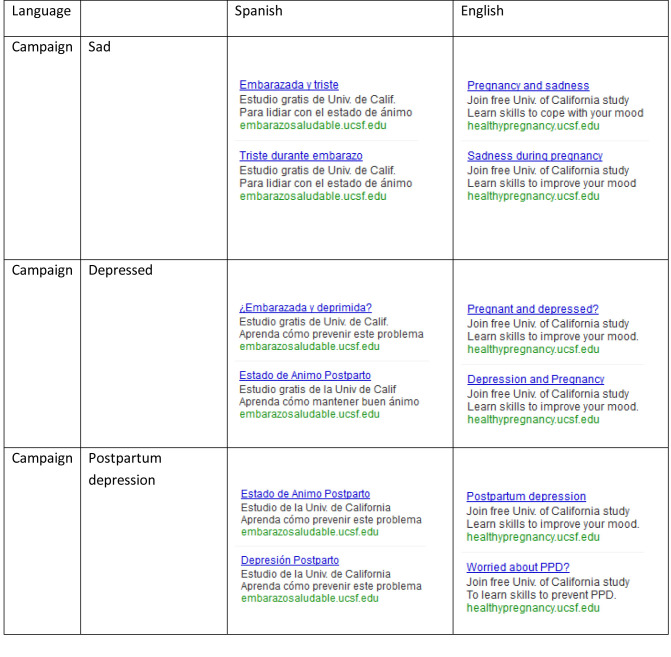
Google ads in Spanish and English for a prevention of postpartum depression trial.

Google AdWords sponsored link campaigns were created in Spanish and English to advertise the Mothers and Babies/*Mamás y Bebés* Internet Project, a RCT to test the efficacy of an Internet intervention for the prevention of PPD. Google ads consist of a 25-character headline and 2 additional 35-character text lines. In this study, the headlines were descriptive of perinatal depression, whereas the text lines described the study and provided the website link. There were 3 ad campaigns (sad, depressed, and postpartum depression) per language, each containing 2 distinctive ads for a total of 6 ads per language (see Figure 1). Ads were distributed worldwide, and the content and associated keywords of each ad remained unchanged throughout the course of the study recruitment period.

The Google AdWords account management website allows the owner of the ads to manipulate the bidding cost and content of each ad. A Google AdWords grant awarded to our team allowed for a maximum bid of US $1.00 per click. Ad owners can view the number of times the ad is shown (impressions) and the number of Web users who click on the ad (clicks); owners can also use HyperText Markup Language (HTML) code to track the impact of the ads on obtaining the desired action by the target audience (conversion). In this study, the HTML code to detect a conversion was integrated into the study website’s consent page (ie, 1 conversion = 1 consent to participate). For the purposes of this paper, we will refer to conversions as “consents” given that it is the desired outcome of online recruitment. Additional information on Google AdWords procedures and metrics can be found elsewhere [38].

### Participant Recruitment

**Figure 2:**
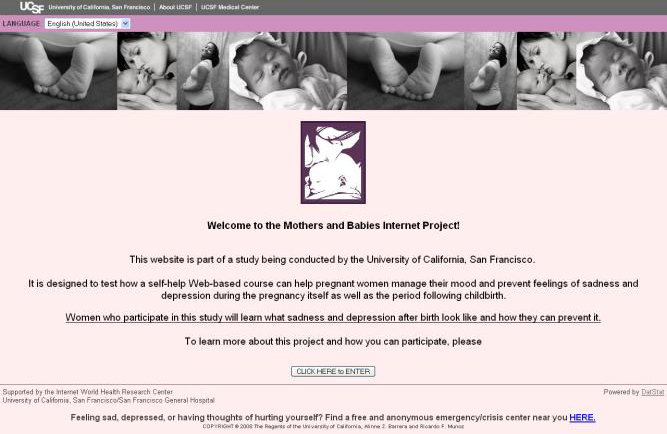
Screenshot of the study landing page.

Web users who searched the Internet between February 3, 2009 and June 15, 2012 with keyword queries associated with the ads were able to click on them and link to the Mothers and Babies/*Mamás y Bebés* Internet Project’s website landing page (Figure 2); this initial page briefly described the study and invited those interested to complete the eligibility screener. The study website was fully automated with items that contained logic to determine participant flow through the study. The eligibility screener contained items to assess for eligibility criteria for the RCT: being female, pregnant, age 18 years or older, and interested in the study website for themselves. Eligible women were directed to the baseline assessment, which contained the University of California institutional review board–approved consent form. Participants “signed” the form online by clicking “Yes, I am interested in participating in this study” and by entering a unique password that was generated in real time and provided online to the user. Eligible participants who consented and completed the baseline assessment were considered enrolled in the study regardless of their depression status or stage of pregnancy. Enrolled participants received access to the intervention sites being evaluated and were invited to complete monthly follow-up assessments up to 6 months postpartum.

### Measures

#### Eligibility Screener

Upon entering the study website, participants indicated their country of residence and preferred language and completed items to determine their eligibility to participate (age, gender, pregnancy status, how they planned to use the website materials). Eligible participants who entered a valid email address were directed to the baseline assessment.

#### Baseline Assessment

The initial page of the survey was the informed consent that participants were required to complete before proceeding through the study website. Consenting participants were asked to complete questionnaire items on demographic characteristics (eg, country of birth, ethnicity, race, education), Internet use (eg, previous use for health information), pregnancy history (eg, weeks pregnant, pregnancy history), and depression (eg, current symptoms).

Depression status was determined by the Center for Epidemiologic Studies-Depression Scale (CES-D) [39] and the Major Depressive Episode (MDE) Screener-Current/Lifetime version [40]. The CES-D is a 20-item self-report instrument that assesses for the presence of depressive symptoms during the past week. Total scores range from 0-60, with higher scores indicating more severe depressive symptoms. The MDE Screener is an 18-item self-report questionnaire that assesses for the presence of 5 or more MDE symptoms experienced within a 2-week or longer period of time during the past 2 weeks (current MDE) or during any period (other than the past 2 weeks) in their lifetime (past MDE). To screen positive for a MDE, significant impairment, as defined by Criterion C of the *Diagnostic and Statistical Manual of Mental Disorders* (Fourth Edition) (*DSM-IV*) must also be present [41]. The MDE Screener is a screening tool that has demonstrated good psychometric properties with diagnostic screeners and clinical interviews [42].

### Data Analysis

Data on the performance of search engine ads were extracted from the Google AdWords management website [38]. Participant data for those who visited (eligibility), consented (started baseline), and enrolled (completed baseline) in the RCT were analyzed using SPSS for Windows 20.0 (IBM Corp, Armonk, NY, USA). Descriptive and chi-square analyses were conducted to examine group differences.

## Results

### Participant Enrollment

Web users interested in the Spanish- and English-language Google ads, as defined by clicking on the ad (ie, clicks), were located in 183 countries and territories; 6745 pregnant women consented to participate with 2517 (37.32%) failing to enter any data in the baseline assessment. Data collected in the eligibility screener indicated that those who consented but did not provide baseline information were older (*P*<.001) and more likely to be English speakers (*P*<.001). Of the remaining 4228 participants, 1653 (24.51% of those who consented) failed to enroll in the intervention study because they did not complete the baseline assessment and, therefore, were excluded from further analyses. The enrolled sample consisted of 2575 participants who met the eligibility criteria, consented to participate, and completed the baseline assessment. Compared to those who consented to participate but did not enroll in the study, participants who enrolled were more likely to be Latino (*P*=.02), older (*P*<.001), employed (*P*=.007), and have attained higher levels of education (*P*<.001). Higher rates of past (11.60% vs 9.51%) and current MDE (19.06% vs 9.69%) were reported for enrolled participants relative to those who did not complete the baseline assessment (*P*<.001).

### Impact of Internet Search Ads

The Google ads were active for approximately 40 months during which 12,983,196 impressions and 176,295 clicks were made in response to Web users’ search entries (see Table 1). In all, 25.09% of those who clicked on the ad entered the study website after reviewing the brief description provided on the study’s landing page. Over 60% of the total impressions (62.27%) were in response to Web users who searched in English, whereas 66.10% of the total clicks were made by those who searched in Spanish. In the Spanish campaign, 116,531 Web users of 4,898,063 who were presented with an ad (ie, an impression) clicked the study ad (2.38%). In the English campaign, there were 59,764 clicks in response to 8,085,133 impressions (0.74%). See Table 2 for detailed data on the performance of each ad campaign by language.

The keywords with the greatest exposure (ie, highest clicks and impressions) and which generated the highest traffic in both languages were pregnancy/*embarazo* and pregnant/*embarazada* (Table 3). For all campaigns, 113,525 clicks of 4,819,662 impressions (2.35%) and 57,197 clicks of 7,836,146 impressions (0.73%) were generated by these keywords in the Spanish and English campaigns, respectively. In addition, those who queried with these keywords were the majority of those who consented to participate—94.53% (4718/4992) and 92.87% (1823/1963), respectively. In contrast, the rate of consent was highest for English-language speakers who searched with keywords that included a reference to both pregnancy and emotions (eg, mental health during pregnancy). A similar pattern was demonstrated in the Spanish campaign with keyword phrases such as “*depresión despues del parto*/depression after birth” or “*embarazada y deprimida*/pregnant and depressed” resulting in a greater proportion of consenting participants. Keywords that were descriptive of pregnancy without a reference to emotions (eg, months pregnant) also resulted in a higher rate of consent. This occurred with more frequency in the Spanish campaign (*nueva mama*/new mother, *meses embarazo*/months pregnant, *semanas embarazo*/weeks pregnant) than in the English campaign (months pregnant).

**Table 1:** Participant online recruitment for the Mothers and Babies/*Mamás y Bebés* Internet Project.

Ad or user behavior	Language, n		Total, n
	Spanish	English	
Impressions (ads presented)	4,898,063	8,085,133	12,983,196
Clicks (clicks on ads)	116,531	59,764	176,295
Entered site (user proceeded beyond initial page)	28,074	16,157	44,231
Screened (user answered ≥1 eligibility items)	11,620	5349	16,969
Eligible (user met eligibility criteria)	8728	3738	12,466
Consented (user agreed to consent)	4773	1972	6745
Enrolled (user consented and completed baseline assessment)	2012	563	2575

**Table 2:** Performance of Google AdWords campaigns from February 3, 2009 to June 15, 2012.

Ad or user behavior	Ad performance, n					
	Sad		Depressed		Postpartum depression	
	Spanish	English	Spanish	English	Spanish	English
Consent	4855	1782	136	179	1	2
Clicks	114,902	53,667	1574	5987	55	110
Impressions	4,794,470	7,184,384	92,280	856,634	11,313	44,115

**Table 3:** Spanish- and English-language ads with the highest-performing keyword(s).

Ad character headline		Keyword(s)	Consents	Cost per consent (US $)	Clicks	Impressions
**Spanish**						
	*Embarazada y triste*	*Embarazo/embarazada* (pregnancy/pregnant)	4718	$15.98	113,525	4,819,662
	*Triste durante embarazo*	*Meses de embarazo* (months pregnant)	156	$8.55	2130	54,987
	*¿Embarazada y deprimida?*	*Depresión en el embarazo* (depression during pregnancy)	109	$2.89	739	11,716
**English**						
	Sadness during pregnancy	Pregnancy/pregnant	1823	$25.77	57,197	7,836,146
	Depression and pregnancy	Pregnancy depression	82	$11.99	1301	97,488
	Pregnancy and sadness	Sad pregnant	17	$12.52	290	18,095
	Pregnant and depressed?	Depressed pregnant	16	$9.12	193	16,457

### Enrolled Participant Characteristics

The final sample of enrolled participants (N=2575) consisted of pregnant women with a mean age of 28.16 years (SD 5.47) (see Table 4). Most completed study materials in Spanish (78.13%), were of Latino/Hispanic ethnic identity (77.89%), and self-identified their racial background as *Mestizo* descent (person of mixed Spanish and Indigenous ancestry; 35.16%) or European descent (30.43%). Most participants were married or living with a partner (66.99%), employed (59.90%), and college educated (70.37%).

There were no differences in pregnancy status or depression history between the Spanish- and English-speaking participants. Participants were mostly in the second trimester of their pregnancy (mean 16.56 weeks, SD 9.59) and most (69.34%) did not meet *DSM-IV* criteria for a MDE. However, more English speakers met the criteria for a current MDE relative to Spanish speakers (20.04% vs 18.81%, respectively), whereas a higher percentage of Spanish speakers met criteria for a past MDE when compared to the English-speaking participants (11.88% vs 10.50%, respectively), although these differences were not statistically significant (*P*=.61). The mean CES-D score was elevated for both samples (mean 27.51, SD 13.77) with Spanish speakers endorsing slightly higher levels of depressive symptoms (*P*=.01). Participants reported accessing the Internet for information related to depression when not pregnant (29.31%) and during the perinatal period (42.64%). Spanish speakers accessed the Internet for depression information at greater proportions than English speakers did, especially when not pregnant or postpartum (32.05% vs 19.35%, *P*<.001).

Group comparisons revealed that the Spanish-speaking women were more likely than English-speaking women to self-identify their ethnic background as Latino or Hispanic (91.45 vs 9.85%, *P*<.001). The racial descent varied significantly (*P*<.001) by language with the Spanish speakers mostly identifying as *Mestizo* (44.10%), European/Caucasian (32.74%), and other (16.42%), whereas the English speakers were of Asian (41.97%), European/Caucasian (21.60%), and African descent (19.75%). Similarly, Spanish speakers resided in Latin America and Spain whereas English speakers were primarily from India (Table 5). A greater proportion of English speakers were married or living with a partner (88.63%) compared to Spanish speakers (60.92%, *P*<.001). English speakers were more likely to have earned advanced educational degrees (28.08% vs 8.75%) whereas Spanish speakers were mostly comprised of college educated (73.38% vs 59.60%, *P*<.001) women.

**Table 4:** Baseline characteristics of enrolled participants.

Demographic items		Spanish n=2012	English n=563	Total N=2575	*P*
Age, mean (SD)		28.18 (5.68)	28.07 (4.60)	28.16 (5.47)	.69
Latino/Hispanic (n=2343,^a^), n (%)		1818 (91.45)	39 (9.85)	1857 (77.89)	<.001
**Race (n=2343), n (%)**					<.001
	*Mestizo* ^b^	819 (44.10)	5 (1.03)	824 (35.17)	
	European/Caucasian descent	608 (32.74)	105 (21.60)	713 (30.43)	
	Other	305 (16.42)	73 (15.02)	378 (16.13)	
	Asian descent	11 (0.59)	204 (41.97)	215 (9.18)	
	African descent	12 (0.65)	96 (19.75)	108 (4.61)	
	American Indian/Alaska Native	102 (5.49)	3 (0.62)	105 (4.48)	
Married/live with partner (n=2572), n (%)		1224 (60.92)	499 (88.63)	1723 (67.99)	<.001
**Education (n=2528), n (%)**					<.001
	12 years or less	353 (17.86)	68 (12.32)	421 (16.65)	
	University level/degree	1450 (73.38)	329 (59.60)	1779 (70.37)	
	Advanced degree	173 (8.75)	155 (28.08)	328 (12.97)	
Employed (n=2561), n (%)		1217 (60.76)	317 (56.81)	1534 (59.90)	.09
Weeks pregnant (n=2564), mean (SD)		16.71 (9.62)	16.01 (9.47)	16.56 (9.59)	.13
**MDE history (n=2492), n (%)**					.61
	None	1371 (69.31)	357 (69.45)	1728 (69.34)	
	Current MDE	372 (18.81)	103 (20.04)	475 (19.06)	
	Past MDE	235 (11.88)	54 (10.50)	289 (11.60)	
CES-D score (n=2475), mean (SD)		27.86 (13.89)	26.07 (13.22)	27.51 (13.77)	.01
**Use Internet for depression information, n (%)**					
	During perinatal period	873 (43.39)	225 (39.96)	1098 (42.64)	.15
	When not pregnant (n=2591)	643 (32.05)	107 (19.35)	750 (29.31)	<.001

^a^Valid percent reflects participants who completed the item.

^b^ *Mestizo*: person of mixed Spanish and Indigenous ancestry.

**Table 5:** Country of residence of enrolled participants.

Country		%^a^
**Spanish-speaking participants (2008/2012)**		
	Chile	16.30
	Mexico	15.24
	Spain	12.00
	Argentina	11.35
	Colombia	11.20
	Venezuela	11.00
	Peru	5.23
	Bolivia	3.43
	Ecuador	2.79
	Dominican Republic	2.54
	Uruguay	1.34
	Paraguay	1.14
	29 Countries/territories with less than 1% each	6.44
**English-speaking participants (562/563)**		
	India	36.30
	South Africa	12.81
	Pakistan	7.47
	United Kingdom	7.12
	Iran	2.67
	Nigeria	2.31
	United States	2.13
	Ghana	1.96
	Kenya	1.78
	United Arab Emirates	1.60
	Saudi Arabia, Maldives, Uganda, Ireland (each)	1.07
	54 Countries/territories with less than 1% each	19.57

^a^Based on self-reported country of residence data.

## Discussion

### Principal Findings

This study examined the impact of Spanish- and English-language Google AdWords to recruit an online sample of participants to an Internet RCT for the prevention of PPD. This method of recruitment was effective at exposing a large number of female Web users to the opportunities offered by the study website, such as learning skills to manage changes in mood during the transition to motherhood. During the 3 years that the ads were active, 176,295 Web users clicked on the advertised link and 12,466 pregnant women met eligibility criteria; 54% agreed to participate and 2575 (20.66%) completed sufficient baseline information to enroll in the study. These data suggest that pregnant women from around the world are interested in learning skills to manage their mood during and after pregnancy, are willing to use Web-based resources, and that the Internet is a viable means to reach them. Although a large number of women showed interest in joining the study, a large proportion exited the site immediately after clicking the ad or failed to continue once they were informed of participation details. A possible explanation for the former is the mismatch between what Web users queried and what they eventually found on the study’s landing page. That is, the information listed on the site did not correspond with what they were looking for or hoping to find when they searched, clicked, and initially visited the study website. The burden of participation may have been a factor to dissuade eligible participants from continuing their engagement in the study once they learned the details of participation from the consent form. Clearly, we need to make such Internet research sites more interesting and less burdensome so that more of those who are eligible not only consent to participate but also complete all facets of the study.

### High Performing Keywords

Our examination of the highest-performing keywords for each of the Google AdWords campaigns revealed 3 primary findings. First, keywords that were more descriptive of the website content (eg, pregnant and depressed) resulted in a higher rate of consent to participate. This pattern of user behavior was evident in both the Spanish and English ads that referenced pregnancy and emotions, and suggests that the biggest gains occur when advertisements closely relate to the product being promoted. This approach is especially important online where there is heavy competition for the user’s attention and sustaining interest in one site over another is challenging. The specificity of pairing a reference to pregnancy and emotions in the keywords resulted in a higher consent rate among Spanish versus English users. This keyword type was also the most cost-effective in both languages relative to other high-performing keyword types (eg, pregnancy without referencing emotions). Cost-effectiveness by itself is not the most important measure of usefulness. The most cost-effective keywords yielded the least number of participants. To recruit the most participants in a reasonable period of time, we must be ready to pay for keywords that yield higher cost per consent. Given that most researchers are working within budgetary constraints, being able to maximize the cost of each consented individual is of high priority and importance. Furthermore, for the English ads there were more variations of this keyword type and, therefore, more related activity. Given that both the Spanish and English ads were identical in their content and associated keywords, this finding may suggest that Spanish-speaking women are less likely to search for information related to emotional health during pregnancy than English-speaking women or that the associated keywords do not reflect how Spanish-speaking women conceptualize perinatal distress. In fact, keywords that did not reference emotions or feelings but described pregnancy characteristics (eg, months pregnant) were very high activity ads and yielded a much larger number of impressions, clicks, consents, and enrolled participants. This suggests that women may be initially attracted by information about pregnancy that does not include information on emotional health during pregnancy. Thus, ads that are more generic may result in better recruitment outcomes for more women, of whom a subset may be subsequently intrigued by the focus on such issues as mood.

### Keyword Language Differences

Previous reports have suggested that Latinos and Spanish speakers manifest psychological distress through physical complaints that can stem from stigma, cultural barriers, and differences in how psychological issues are conceptualized and experienced [43,44]. This study found that ads associated with broad search terms (eg, pregnancy) had greater reach and enrollment impact. This pattern of user behavior was evident in both languages and is consistent with female patterns of online search behavior which shows that women, more than men, search for health-related issues and that pregnancy-related queries are a major topic of inquiry among childbearing-aged female Web users [45,46]. There is a high likelihood that ads that were linked to broad keywords appeared in a large number of searches, many of which did not result in a visit to the study website. However, Spanish-speaking women who used broad keywords in their queries appeared to be more interested in the ads than English-speaking women as evidenced by the higher number of clicks and consents relative to the number of impressions which were almost twice as high in the English ads. We speculate that there may be fewer such Internet resources in Spanish than in English, therefore drawing a higher number of Spanish speakers to our website. These data indicate that broad, nonspecific keywords were effective at recruiting a large, diverse sample of pregnant women. However, this approach was more costly in the long term than more-specific keywords, which were more cost-effective per consent but resulted in fewer consenting participants. Researchers need to consider what their recruitment goals are and adjust their ad campaigns accordingly. To the best of our knowledge, this is the first fully automated prevention of PPD trial to recruit a large worldwide sample of Spanish- and English-speaking pregnant women. At the start of our recruitment period, we were uncertain of the potential interest of the study among pregnant women. Thus, our initial recruitment goal, which was to reach a large number of potential female Web users across the world, was accomplished.

### Participant Characteristic Outcomes

A secondary aim of this study was to examine the characteristics of individuals who consented to participate in a Web-based trial. The ads attracted a mostly Spanish-speaking sample of women who resided in Latin American countries. This was an unexpected outcome of the recruitment efforts given the lower Internet penetration rate in this region of the world relative to Oceania/Australia, Europe, and North America [47]. Although the English-speaking sample was smaller, participants were equally diverse with the largest number of participants indicating that they resided in India and across different regions of Africa. The geographic location of participants mirrors the recent gender shift in Internet access by women in developed and developing nations. Furthermore, it highlights the potential need to develop new technology-based interventions targeting women from all around the world, especially those with current limited access to online resources but whose access is growing at a rapid rate (eg, 1310.8% growth in Latin American and the Caribbean in the past 10 years) [47].

Spanish- and English-speaking participants were relatively similar on demographic characteristics with a few exceptions. The breakdown of the origin of our participants is important within a global mental health perspective, and especially critical when considering maternal mental health needs worldwide. To date, few resources exist in these regions of the world to address the day-to-day needs of pregnant women [48,49], especially as they relate to mental health issues during pregnancy. Furthermore, these women are accessing the Internet in regions of the world where the overall Internet penetration is lower and men significantly outnumber women in their use of the Internet [11,50]. This gender divide is likely to change as the global use of technology continues to grow.

Participants in this study were well educated, with a greater proportion of English-language speakers possessing advanced degrees, whereas Spanish-language speakers had mostly attended university level or fewer years of formal education. It is clear that women with varied levels of education are using the Internet to obtain pregnancy-related information. Spanish- and English-speaking participants differed in their marital status with Spanish speakers more likely to indicate that they were single or without a current partner, thus potentially raising their risk for the onset of postpartum mood disorders [51]. Finally, the high rate of major depression found in this sample is of clinical concern and echoes a recent call-to-action to make maternal mental health a top priority [52]. Nearly one-third (30.66%) of our participants screened positive for a MDE sometime in their lifetime, yet our recruitment efforts did not seek or select participants based on their depression status. This indicates that the Internet is an effective tool to reach pregnant women who are depressed or at high risk for depression. Rates of perinatal depression range from 10%-25% [53,54], with point prevalence rates during pregnancy hovering around 12%-15% [51,55]. The higher rate of MDE found in this study may be attributed to measurement differences, the use of a self-report screening measure and not on clinician diagnostic information, the symptom overlap between pregnancy and depression, or to self-selection. Regardless, high rates of depressive symptoms were reported which is consistent with online samples of postpartum women [22]. These data highlight the need to develop multilingual, culturally considerate Internet resources for pregnant women regardless of depression risk. Many websites provide pregnancy information and many pregnant women are accessing the Internet to help guide their prenatal and postpartum health care [56]. However, the quality and reliability of the sites that include perinatal mental health topics is variable with a majority falling short of relaying complete and accurate information and even fewer providing practical skills to reduce symptoms [18]. Current studies that are in progress, such as the Mothers and Babies/*Mamás y Bebés* Internet Project directed by our team, as well as other prevention of PPD trials [27,28], stand to make a significant contribution to the availability of empirically tested Internet interventions to prevent PPD.

### Limitations

Our findings were limited in several ways. First, the Google AdWords grant awarded to our team capped our bids at US $1.00. Second, this study solely reports on the use of minimally modified Google AdWords campaigns. We do not know how the ads would have performed if we had managed the campaigns based on their performance by adjusting the keywords, daily cost, or regional exposure. Third, given how the ads were set up and linked to the study website, we are unable to connect keyword types to participant characteristics. In order to understand how to target the ads to participants in different regions of the world who speak different languages and who conceptualize emotional distress differently, it would be beneficial to explore this further by recording the keyword entered by the user in their online search and examining their characteristics and behaviors on the study website. Finally, our data are only generalizable to Spanish and English speakers who use the Web to obtain information on the perinatal period.

### Conclusions

This study contributes to the growing understanding of online recruitment for intervention trials. We provide evidence that pregnant women in many regions of the world are already seeking this type of health information and choosing to engage in Internet interventions by virtue of their participation in this study. Nonconsumable Internet interventions or those that can be reused with minimal added cost have the potential to reduce health disparities globally because they can be used an unlimited number of times from any region of the world without significant increases in cost [1]. The opportunity to share these resources with a wide range of individuals who may lack local resources requires the ability to design and test these interventions with global samples of individuals from diverse ethnic and cultural backgrounds. The health field would do well to use the potential of Internet interventions to increase health resources focused on maternal mental health and to reduce health disparities where there are few resources to protect childbearing women and advance the well-being of mothers and their babies.

## Abbreviations

CES-D: Center for Epidemiological Studies-Depression
HTML: HyperText Markup Language
MDE: major depressive episode
PPD: postpartum depression
RCT: randomized controlled trial
